# Temporally Dissociated, Trait-Specific Modifications Underlie Phenotypic Polyphenism in *Spea multiplicata* Tadpoles, Which Suggests Modularity

**DOI:** 10.1100/tsw.2007.159

**Published:** 2007-05-29

**Authors:** Brian L. Storz, Joseph Travis

**Affiliations:** Department of Biological Science, Florida State University, Tallahassee, Florida, USA

**Keywords:** Spea, spadefoot toad, modularity, development, heterochrony, allometry

## Abstract

Many organisms that develop in a variable environment show correlated patterns of phenotypic plasticity in several traits. Any individual trait modification can be beneficial, neutral, or deleterious in any particular environment; the organism's total fitness, which determines if the plasticity is adaptive, is the sum of these changes. Although much is known about how plastic traits contribute to fitness, less is known about the extent to which the various trait changes involved in the plastic responses share their developmental control. Shared control suggests that the various responses evolved in unison, but independent control suggests independent evolution of many components. Spadefoot toads have evolved adaptive polyphenism to cope with developing in rapidly drying ephemeral ponds. Larvae hatch as omnivores, but on exposure to an environmental cue, may develop into carnivores. We compared trait development in the two morphs and found that differences in jaw musculature, head dimensions, and intestines emerged early in development, whereas differences in shape of the tail emerged later. In omnivores, all traits except intestine length and hind-limb length were negatively allometric with body length; in carnivores, two of three jaw muscles displayed positive allometry and, among those that were negatively allometric, all except head width showed larger allometric coefficients in carnivores. Hind-limb length was positively allometric in both forms, but the allometric coefficients did not differ significantly. Intestine length was positively allometric to body length in both forms, but in this case, omnivores exhibited the higher coefficient. These results suggest that spadefoot plasticity is trait specific and the responses are suggestive of the existence of at least two modules: a suite of trophic traits that responds early in development and a suite of tail traits that responds later. The developmental control of these suites is the subject of further investigation.

## INTRODUCTION

Developmental plasticity, the ability to produce multiple phenotypes from one genotype, has long been of interest to biologists[1,2]. Its adaptive importance lies in the organism's ability to perceive characteristics of the environment and, in response, to switch to a developmental pathway that will yield a phenotype better suited to that environment[3]. Developmental plasticity should be favored in fluctuating environments in which a reliable cue signals approaching change[3,4,5]. Some of the best-known examples include plant shade avoidance in response to high density[6]; alternate caterpillar morphology induced by seasonal diet differences[7]; and tadpole plasticity for larval period, morphology, and behavior in response to pond duration, temperature, food level, and predation risk[8,9,10,11,12,13,14, 15,16,17,18,19].

Studies of plasticity have shown that several traits are usually modified in a correlative manner in response to an environmental cue. For example, male sailfin mollies (Poeciliidae: *Poecilia latipinna*) that experience higher temperatures have a lower visceral mass for their size, but a higher testis mass[20], individual *Daphnia pulex* respond to predation risk by altering several aspects of their morphology and life history[21], and changes in tadpole tail morphology in response to predators usually involve changes in several individual traits[17,19,22]. When several traits change in response to an environmental cue, each individual trait modification may be beneficial, neutral, or deleterious in any particular environment, and the organism's total fitness is the sum of these effects[23,24]. Plasticity, therefore, must be understood as alteration of entire developmental trajectories and not just as putatively adaptive shifts in a few traits[25]. If this is so, then it is important to understand how many developmental trajectories are involved, i.e., to understand if all of the traits have been molded to respond independently by selection for plasticity, whether there are interconnected units that change relatively independently of other interconnected units[26,27] or whether the individual traits are so tightly constrained by shared control into suites that they exhibit very few possible discrete plastic responses.

New World spadefoot toads are an especially suitable system for studying developmental integration of plasticity and its consequences. Larvae of at least two species (*Spea multiplicata* and *S. bombifrons*) show a striking polyphenism between "typical" filter-feeding omnivores and carnivores (Fig. 1) that actively prey on microcrustaceans and conspecifics. The phenotypes are so dissimilar that they were originally classified as different subspecies[28]. This polyphenism is thought to have evolved as an adaptation for survival in temporary pond environments[14,29]. In these environments, survival depends on the ability to develop and metamorphose rapidly. Carnivorous individuals have been shown to have a competitive advantage in rapidly drying ponds because they metamorphose sooner than omnivores[30], thereby avoiding desiccation. Conversely, in long-lived ponds, omnivores have higher survival at metamorphosis because of their greater fat reserves[14,29].

*Spea multiplicata* tadpoles hatch as omnivores, but on exposure to an environmental cue[14], individuals may shift their ontogenies to become carnivores. Carnivores are known to have enlarged heads, enlarged jaw musculature, shortened intestines, and increased keratinization of the mouth to form a beak, relative to omnivores (Figs. 1A–C)[14,29,30,31]. However, it is not known whether these traits change independently or as developmental modules, whether the change from omnivore to carnivore represents a shift in total somatic growth or only in specific traits, or whether developmental trait modification has additional, as yet unknown, consequences.

In this study, we address these questions as a first step toward investigating the developmental trajectories that produce these strikingly different morphs. We ask whether the change from omnivore to carnivore represents a shift in total somatic growth or only in specific traits, whether these traits change independently or concordantly, and whether we can identify inherent trade-offs among traits associated with becoming a carnivore. We present a morphological analysis of developmental polyphenism in *S. multiplicata*, showing that the omnivore developmental program is broken up and modified in a trait-specific manner to produce the carnivore phenotype, and trait modifications are grouped and occur at different developmental stages, suggesting trait modularity and at least two levels of shared developmental control.

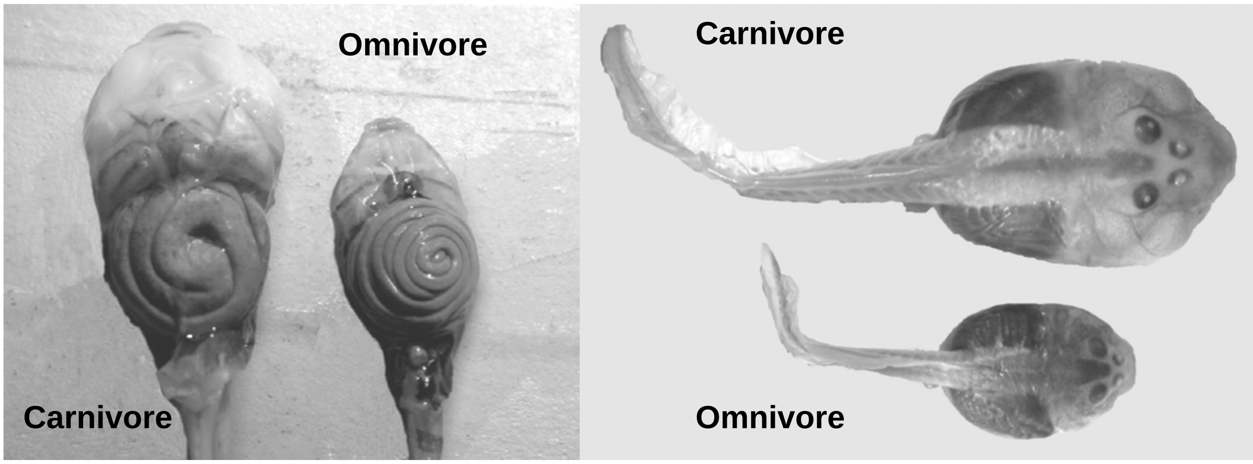

**A**

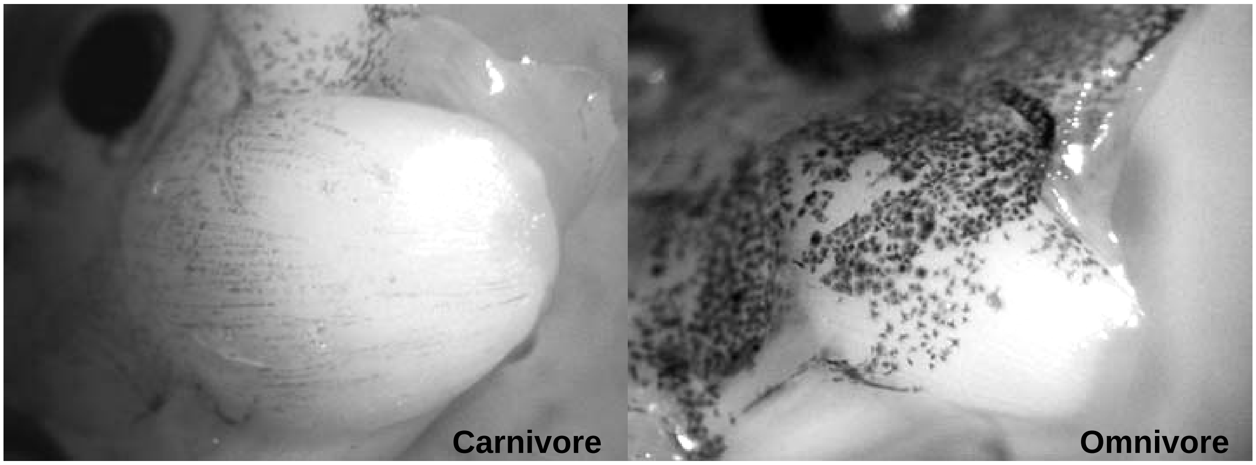

**B**

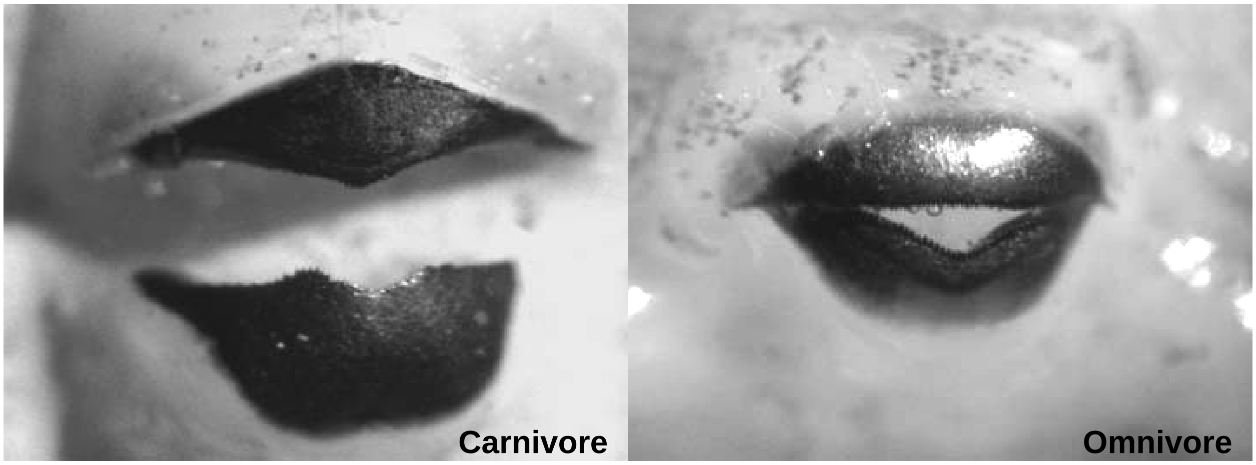

**C**

**FIGURE 1**. *Spea multiplicata* phenotypes shown are at Gosner stage 36 (middle hind-limb development), at which phenotypes show their greatest differences in size and morphology. Size and shape differences between the phenotypes are apparent in intestines, jaw musculature, and beak morphology. (A) Carnivores have wider and shorter intestines than do omnivores, (B) carnivore orbitohyoideus jaw muscles show hypertrophy relative to those of omnivores, and (C) carnivores have more sharply pointed beaks than do omnivores.

## MATERIALS AND METHODS

Naturally developing *S. multiplicata* omnivores and carnivores were collected in July and August 2002 over a period of 25 days at developmental stages 27–42 from Dearing Pond, approximately 1.6 km north of Portal Road, Cochise County, Arizona (see Table 1 for sample sizes of each morph at each stage). Only one breeding aggregation occurred at this pond; all eggs were laid on the same evening and the cohort of tadpoles could be followed throughout development in a natural setting. Tadpoles were collected by dip net or seine, and sorted according to phenotype and Gosner stage[32]. Phenotype was easily determined by inspection of the beak morphology (Fig. 1C). Larvae were euthanized with an overdose of MS-222 and preserved in 10% formalin. All morphological measurements were taken, in millimeters, with an ocular micrometer or by digital image analysis (Image J NIH version 1.29X).

**TABLE 1**
**Number of Carnivores and Omnivores at Each Gosner Developmental Stage Measured for Analyses**

| | Gosner Developmental Stage | | | | | | | | | | | | | |
|---|---|---|---|---|---|---|---|---|---|---|---|---|---|---|
| | **27** | **28** | **29** | **30** | **31** | **32** | **34** | **35** | **36** | **38** | **39** | **40** | **41** | **42** |
| Carnivore | 3 | 12 | 10 | 11 | 10 | 9 | 6 | 9 | 11 | 10 | 11 | 8 | 10 | 9 |
| Omnivore | 17 | 9 | 9 | 9 | 7 | 0 | 10 | 7 | 10 | 13 | 10 | 10 | 10 | 11 |

In addition to snout-to-vent length (SVL)[33], we recorded averaged width of the two orbitohyoideus jaw muscles, two measures of the interhyoideus width (measured at the widest point [as by 14,29,30]), averaged width of the two hyoangularis muscles, mouth width, head width (as a straight-line measurement of interhyoideus length), head length (as a straight-line measurement from snout to mid-eye), tail length (from cloaca to the posterior tip of the tail muscle), tail-fin height (just posterior to the cloaca), tail-muscle height (just posterior to the cloaca), hind-limb length (from point of attachment to terminal end of footpad), and intestine length (the distance, measured on intestines dissected from tadpoles, from the beginning of the mid-gut to the end of the rectum, at point of attachment to the cloaca). Omnivore and carnivore phenotypes show radically different trait growth curves when untransformed measurements are plotted against SVL; thus, to standardize growth curves for purposes of direct comparison, all trait measurements were log (base 10) transformed for all analyses.

We used Gosner stages 27–42 for carnivore and omnivore trait disparity analysis, but only stages 27–40 in linear allometry and discriminant function analyses. Later developmental stages were excluded from these analyses because metamorphic climax begins just after Gosner stage 40, and larval phenotype differences due to polyphenism could be confused with those due to metamorphic climax. Hind-limb length was also excluded from discriminant function analysis because the relatively large mean and variance may obscure the importance of other traits in distinguishing omnivore and carnivore phenotypes, and no significant allometric difference was found between carnivore and omnivore phenotypes.

We determined the developmental stage at which trait disparity between the phenotypes originated and how long it was maintained during larval ontogeny by *t*-test of log (base 10)-transformed trait measurements on the two phenotypes at each Gosner developmental stage (27–42). Although the stage analyses were based on some 143 *t*-tests, we deemed Bonferroni corrections unnecessary because the results at each stage are independent; they are based on different animals collected on different days (independent collections). We used simple, linear allometry of the log-transformed trait measurements against SVL to investigate trait growth differences between the phenotypes and analysis of covariance to test for significance of slope difference. We used discriminant function (DF) step-down analysis[34] to determine which trait(s) contributed the most to the differences between the two phenotypes. In the DF

analysis, we calculated a test statistic for the multivariate differences (Wilks' lambda) using all carnivore and omnivore traits; we calculated an F-statistic for each trait (F-to-remove) to determine whether the phenotypes differed in each trait, allowing for the effects of the other traits. We removed the trait with the lowest F statistic and recalculated the Wilks' lambda using the remaining traits. This iterative procedure retains those traits that provide the best discrimination between the phenotypes. We used log data rather than size-adjusted data in the DF analysis because one of the questions we sought to answer was "how do the phenotypes differ?"; size is an element in this analysis and the DF analysis examines the role of each trait independently of the other traits, including size.

## RESULTS

### Developmental Timing

All tadpoles hatch with the omnivore phenotype, and phenotypes are indistinguishable at posthatching stages 25 and 26[31]. Trait differences between the morphs emerged at two distinct times in development (Table 2, Fig. 2). Carnivores exhibited larger jaw musculature, larger heads, and shorter intestines at the earliest stage we examined (Gosner stage 27, approximately 3 days after hatching), and the difference persisted throughout larval development. The magnitude of the difference increased only slowly during subsequent development. The larger tails of carnivores did not appear until later in development (Gosner stage 34, approximately 10 days after hatching), and the discrepancy between the morphs increased substantially as development progressed. Carnivore and omnivore phenotypes later converge during metamorphic climax and are indistinguishable on emergence from ephemeral ponds; the beginning of convergence is graphically illustrated by the shift towards zero of the intestine length and jaw musculature in Fig. 2.

### Allometry

With the exception of intestine length and hind-limb length, all omnivore traits showed negative allometry with body length. In contrast, traits in carnivores showed a mixture of positive and negative allometry and isometry (Table 3). Traits involved in the trophic apparatus (mouth width and jaw musculature as measured by interhyoideus width, orbitohyoideus width, and hyoangularis width) and tail structure (tail length, tail-fin height, tail-muscle height) had steeper slopes when plotted against SVL in carnivores than in omnivores. Two traits, head length and intestine length, had steeper slopes in omnivores than in carnivores, but after we applied Dunn-Sidak correction to adjust for multiple slope tests, the difference in the slope of head length was no longer significant. Finally, in two traits, head width and hind-limb length, the phenotypes did not differ (Figs. 3A–D, Table 3). Because these data are drawn from a developmental series, steeper slopes represent traits that are growing faster relative to body length (SVL) in one or the other phenotype. Therefore, traits tightly connected to the mode of tadpole feeding and locomotion grew faster in carnivores than in omnivores, whereas some traits grew more slowly and others grew at the same rates in the two phenotypes.

### Discriminant Function Analysis

Discriminant function analysis identified intestine length (F-to-remove = 97.86, Wilks' lambda $F_2$ = 2293.55, $p$ = 0.00) and interhyoideus width (F-to-remove = 40.59, Wilks' lambda $F_2$ = 2293.55, $p$ = 0.00) as the traits that best discriminate between the phenotypes. The prominence of these traits is due to three- to fourfold longer intestines in omnivores and massive enlargement of carnivore jaw musculature relative to omnivores, even at the earliest stages of development (Gosner stage 27, presence of hind limb buds).

**TABLE 2**
***T*-Test of Log (Base 10)-Transformed Trait Measurements at Each Gosner Developmental Stage**

| Trait | Gosner Developmental Stage | | | | | | | | | | | | |
|---|---|---|---|---|---|---|---|---|---|---|---|---|---|
| | **27** | **28** | **29** | **30** | **31** | **34** | **35** | **36** | **38** | **39** | **40** | **41** | **42** |
| Jaw muscles | | | | | | | | | | | | | |
| Interhyoideus width | $T_{18}$ = 9.48 ** | $T_{19}$ = 7.23 ** | $T_{17}$ = 6.72 ** | $T_{18}$ = 11.03 ** | $T_{15}$ = 9.12 ** | $T_{14}$ = 7.66 ** | $T_{14}$ = 11.29 ** | $T_{19}$ = 28.99 ** | $T_{21}$ = 17.42 ** | $T_{19}$ = 23.04 ** | $T_{16}$ = 25.49 ** | $T_{18}$ = 21.76 ** | $T_{18}$ = 10.18 ** |
| Orbitohyoideus width | $T_{18}$ = 9.62 ** | $T_{19}$ = 7.24 ** | $T_{17}$ = 6.75 ** | $T_{18}$ = 10.81 ** | $T_{15}$ = 8.51 ** | $T_{14}$ = 9.88 ** | $T_{14}$ = 15.10 ** | $T_{19}$ = 49.47 ** | $T_{21}$ = 17.36 ** | $T_{19}$ = 17.74 ** | $T_{16}$ = 18.98 ** | $T_{18}$ = 19.15 ** | $T_{18}$ = 12.62 ** |
| Hyoangularis width | $T_{18}$ = 5.80 ** | $T_{19}$ = 6.97 ** | $T_{16}$ = 7.89 ** | $T_{18}$ = 11.32 ** | $T_{15}$ = 9.32 ** | $T_{14}$ = 9.43 ** | $T_{14}$ = 11.51 ** | $T_{19}$ = 40.36 ** | $T_{21}$ = 17.63 ** | $T_{19}$ = 16.21 ** | $T_{16}$ = 21.51 ** | $T_{18}$ = 17.11 ** | $T_{18}$ = 7.204 ** |
| Intestine length | $T_{18}$ = 6.36 ** | $T_{19}$ = 5.41 ** | $T_{17}$ = 9.04 ** | $T_{17}$ = 23.66 ** | $T_{18}$ = 18.75 ** | $T_{18}$ = 6.83 ** | $T_{18}$ = 9.71 ** | $T_{15}$ = 24.32 ** | $T_{17}$ = 17.50 ** | $T_{17}$ = 23.56 ** | $T_{17}$ = 22.56 ** | $T_{17}$ = 22.56 ** | $T_{17}$ = 9.89 ** |
| **Head traits** | | | | | | | | | | | | | |
| Head width | $T_{18}$ = 1.79 ns | $T_{19}$ = 4.72 * | $T_{17}$ = 4.18 * | $T_{18}$ = 7.58 ** | $T_{15}$ = 6.45 ** | $T_{14}$ = 5.54 ** | $T_{14}$ = 10.84 ** | $T_{19}$ = 39.95 ** | $T_{21}$ = 18.82 ** | $T_{19}$ = 17.69 ** | $T_{16}$ = 20.86 ** | $T_{18}$ = 17.75 ** | $T_{18}$ = 11.58 ** |
| Head length | $T_{18}$ = 2.40 * | $T_{19}$ = 5.12 ** | $T_{17}$ = 3.93 * | $T_{18}$ = 7.35 ** | $T_{15}$ = 6.15 ** | $T_{14}$ = 6.73 ** | $T_{14}$ = 13.49 ** | $T_{19}$ = 31.87 ** | $T_{21}$ = 14.43 ** | $T_{19}$ = 18.99 ** | $T_{16}$ = 23.42 ** | $T_{18}$ = 17.56 ** | $T_{18}$ = 8.20 ** |
| Mouth width | $T_{18}$ = 1.23 ns | $T_{19}$ = 4.10 * | $T_{16}$ = 2.41 * | $T_{18}$ = 3.26 * | $T_{15}$ = 2.10 * | $T_{14}$ = 2.52 * | $T_{14}$ = 5.12 * | $T_{19}$ = 13.40 ** | $T_{21}$ = 6.52 ** | $T_{19}$ = 7.79 ** | $T_{16}$ = 11.68 ** | $T_{18}$ = 6.29 ** | $T_{18}$ = 6.88 ** |
| **Tail traits** | | | | | | | | | | | | | |
| Tail length | $T_{13}$ = 1.57 ns | $T_{13}$ = 4.06 * | $T_{14}$ = 0.63 ns | $T_{13}$ = 0.46 ns | $T_{12}$ = 1.20 ns | $T_{11}$ = 2.48 * | $T_{15}$ = 5.91 ** | $T_{16}$ = 8.90 ** | $T_{15}$ = 9.77 ** | $T_{17}$ = 12.83 ** | $T_{15}$ = 12.31 ** | $T_{15}$ = 7.64 ** | $T_{16}$ = 4.44 * |
| Tail-fin height | $T_{18}$ = 0.13 ns | $T_{19}$ = 0.23 ns | $T_{14}$ = 1.53 ns | $T_{18}$ = 1.85 ns | $T_{15}$ = 1.01 ns | $T_{14}$ = 2.79 * | $T_{14}$ = 4.83 * | $T_{19}$ = 5.60 ** | $T_{21}$ = 5.78 ** | $T_{19}$ = 9.78 ** | $T_{16}$ = 9.53 ** | $T_{18}$ = 7.42 ** | $T_{18}$ = 4.62 * |
| Tail-muscle height | $T_{18}$ = 1.24 ns | $T_{19}$ = 2.05 * | $T_{16}$ = 0.76 ns | $T_{18}$ = 0.25 ns | $T_{15}$ = 1.87 ns | $T_{14}$ = 3.44 * | $T_{14}$ = 6.14 ** | $T_{19}$ = 8.07 ** | $T_{21}$ = 13.19 ** | $T_{19}$ = 14.63 ** | $T_{16}$ = 9.41 ** | $T_{18}$ = 11.62 ** | $T_{18}$ = 8.92 ** |
| SVL | $T_{18}$ = 0.045 ns | $T_{19}$ = 1.69 ns | $T_{17}$ = 0.08 ns | $T_{18}$ = 1.73 ns | $T_{15}$ = 0.13 ns | $T_{14}$ = 1.91 ns | $T_{14}$ = 5.99 ** | $T_{19}$ = 13.73 ** | $T_{21}$ = 9.67 ** | $T_{19}$ = 13.19 ** | $T_{16}$ = 16.52 ** | $T_{18}$ = 11.80 ** | $T_{18}$ = 9.73 ** |

*Note:* Carnivores show significantly larger jaw muscles and head traits, and smaller intestines just after hatching, whereas tail traits and SVL are not significantly larger in carnivores until mid-development.

* Significant at ≤0.05.

** Significant at <0.0001 level.

These trait differences can be visualized in a graph of log intestine length against log interhyoideus width, including measurements during metamorphic climax, although these measurements were not included in the discriminant function analysis (see methods). Fig. 4 clearly shows that the phenotypes occupied different regions of morphospace during larval ontogeny, but converge at metamorphic climax.

## DISCUSSION

Carnivore and omnivore phenotypes were fundamentally dissimilar and demonstrated differences in size, shape, and multivariate trait organization. The omnivore developmental program was modified to produce the carnivore phenotype by trait-specific growth rate changes, and although most traits in omnivores were negatively allometric, carnivores showed a mixture of negative allometry, positive allometry, and isometry.

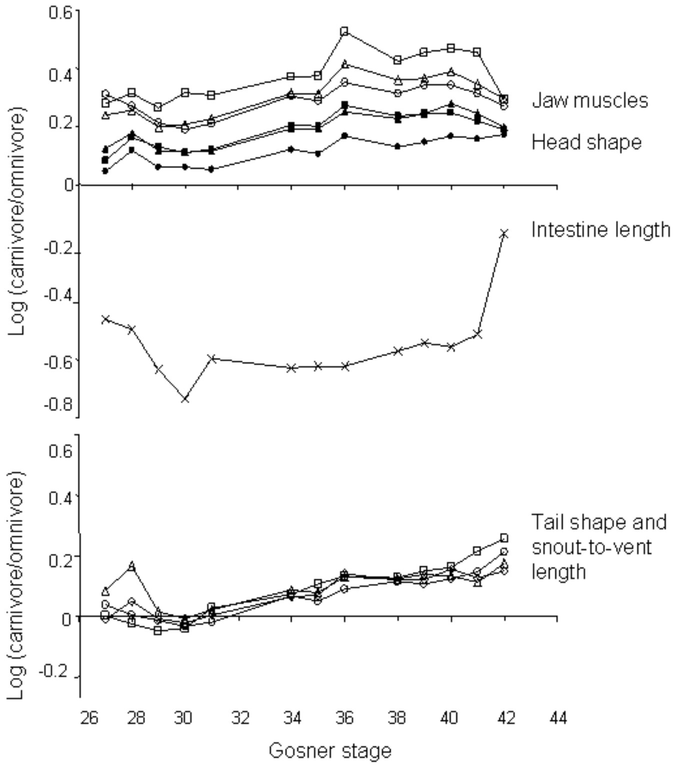

**FIGURE 2**. Log (carnivore trait/omnivore trait) as a function of Gosner developmental stage. Means are represented. Jaw musculature (upper panel: hyoangularis, open squares; orbitohyoideus, open triangles; interhyoideus, open circles), head shape (upper panel: head length, filled triangle; head width, filled square; mouth width, filled circle), and intestines (upper panel: x symbol) show divergence at the earliest developmental stages, whereas tail shape (lower panel: tail length, open triangles; tail-muscle height, open circles; tail-fin height, open squares) and SVL (lower panel: open diamonds) show divergence at mid-larval period.

This finding is remarkable because it suggests that the larval developmental program can be broken up and modified at individual tissues, but that the developmental program is still coordinated enough to produce a postmetamorphic, juvenile toad.

Also striking is that these traits are modified, at least statistically, as two distinct groups, suggesting trait modularity. Researchers have argued for the existence of developmental modules on the basis of multiple types of evidence, including genetic and phenotypic trait covariance and conservation of developmental series[35,36,37]. In the study reported here, we suggest the existence of modularity because of differences in timing of correlated trait shifts. For example, carnivore trophic structure diverged from that of omnivores at the earliest stages of development, whereas carnivore tail traits were modified at mid-larval period. Further support for the existence of two developmental modules in this system comes from both preliminary histological studies of skeletal muscle enlargement and embryological studies of skeletal muscle in other systems. Our preliminary enlargement analyses of both the orbitohyoideus jaw and tail muscles — which are representative traits of the early and late modules, respectively — in carnivores relative to omnivores shows that hypertrophy (an increase in myofiber size) underlies enlargement of the orbitohyoideus jaw muscle, while a combination of hyperplasia (an increase in myofiber number) and hypertrophy is responsible for tail muscle enlargement. Furthermore, embryological studies of skeletal muscle in other systems have shown that cranial musculature originates from somitomeres while trunk musculature originates from mature somites and that Pax 3 helps to regulate myoblast determination in trunk

**TABLE 3**
**Linear Regressions of Log-Transformed Traits on Log SVL and Results of ANCOVA Comparing Slopes of Traits Plotted Against SVL in the Carnivore and Omnivore Phenotypes of Spadefoot Toads**

| | Slope ± S.E. | $R^2$ | ANCOVA | |
|---|---|---|---|---|
| | | | *F* Ratio | *p* Value |
| Jaw musculature | | | | |
| Interhyoideus width | | | 18.77 | <0.0001* |
| Carnivore | 0.97 ± 0.001 | 0.94 | | |
| Omnivore | 0.75 ± 0.004 | 0.74 | | |
| Orbitohyoideus width | | | 75.11 | <0.0001* |
| Carnivore | 1.07 ± 0.001 | 0.94 | | |
| Omnivore | 0.69 ± 0.003 | 0.80 | | |
| Hyoangularis width | | | 49.45 | <0.0001* |
| Carnivore | 1.15 ± 0.003 | 0.89 | | |
| Omnivore | 0.71 ± 0.005 | 0.68 | | |
| Head shape | | | | |
| Head width | | | 1.78 | 0.18 |
| Carnivore | 0.96 ± 0.0008 | 0.95 | | |
| Omnivore | 0.92 ± 0.0006 | 0.97 | | |
| Head length | | | 4.02 | 0.05 |
| Carnivore | 0.92 ± 0.0006 | 0.96 | | |
| Omnivore | 0.97 ± 0.0009 | 0.96 | | |
| Mouth width | | | 9.38 | 0.002* |
| Carnivore | 0.85 ± 0.001 | 0.91 | | |
| Omnivore | 0.69 ± 0.004 | 0.71 | | |
| Tail structure | | | | |
| Tail length | | | 14.27 | 0.0002* |
| Carnivore | 0.99 ± 0.0006 | 0.96 | | |
| Omnivore | 0.79 ± 0.005 | 0.76 | | |
| Tail-fin height | | | 60.61 | <0.0001* |
| Carnivore | 0.92 ± 0.002 | 0.85 | | |
| Omnivore | 0.51 ± 0.003 | 0.64 | | |
| Tail-muscle height | | | 20.66 | <0.0001* |
| Carnivore | 0.93 ± 0.001 | 0.91 | | |
| Omnivore | 0.70 ± 0.004 | 0.72 | | |
| Other | | | | |
| Hind-limb length | | | 0.39 | 0.53 |
| Carnivore | 3.01 ± 0.01 | 0.91 | | |
| Omnivore | 2.86 ± 0.07 | 0.60 | | |
| Intestine length | | | 11.99 | 0.0006* |
| Carnivore | 1.24 ± 0.006 | 0.80 | | |
| Omnivore | 1.60 ± 0.02 | 0.79 | | |

*Note:* All regressions are significant at $p < 0.0001$ and ANCOVA (*) at 0.05. Traits involved in the trophic apparatus and tail structure had steeper slopes when plotted against SVL in carnivores than in omnivores.

* Significant at 0.05 level with Dunn-Sidak correction for multiple comparisons.

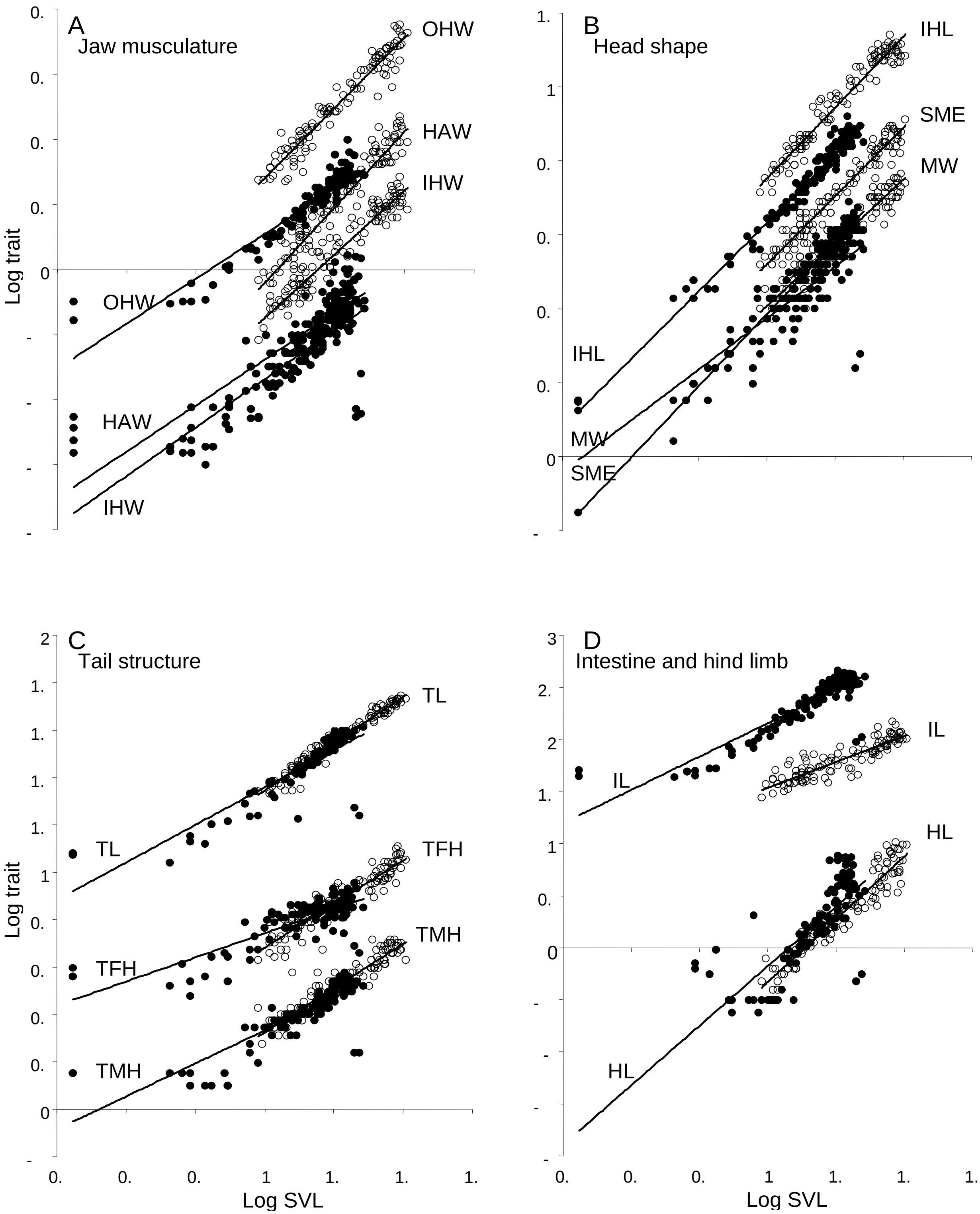

**FIGURE 3**. Simple linear regression of log-transformed traits on log SVL for carnivores (open symbols) and omnivores (filled symbols). (A) Jaw-musculature measurements, (B) head measurements, (C) tail measurements, (D) intestine length and hind-limb length. Large differences are apparent in jaw musculature, tail morphology, and intestine development. OHW, orbitohyoideus width; HAW, hyoangularis width; IHW, interhyoideus width; IHL, interhyoideus length; SME, head length; MW, mouth width; TL, tail length; TFH, tail-fin height; TMH, tail-muscle height; IL, intestine length; HL, hind-limb length.

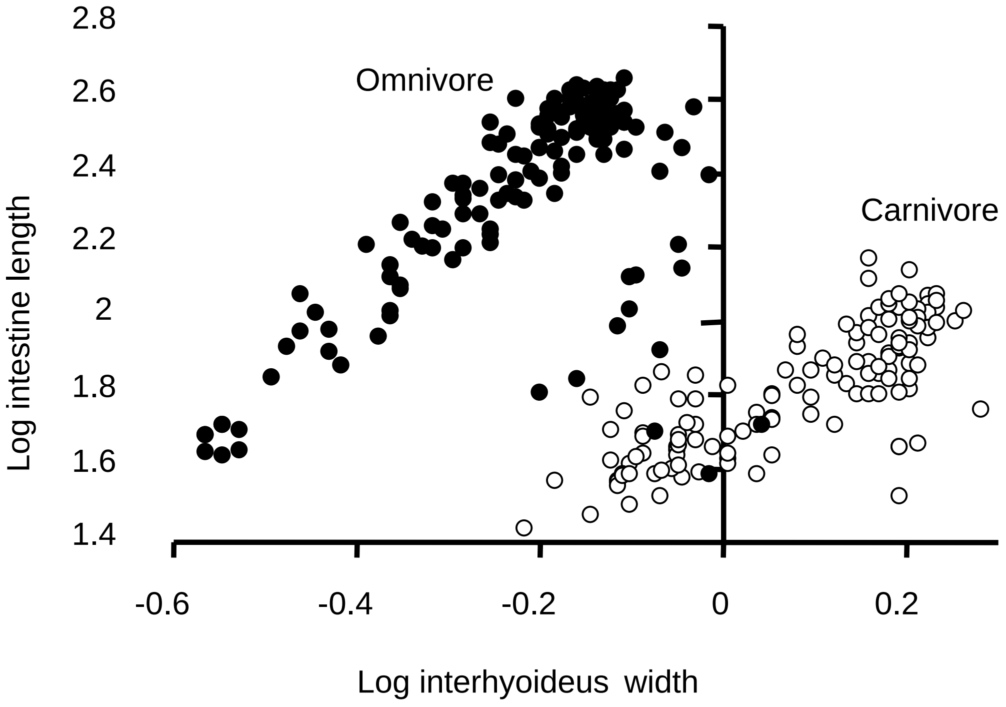

**FIGURE 4.** Log intestine length plotted against log interhyoideus width for carnivores (open symbols) and omnivores (filled symbols). Carnivore and omnivore phenotypes are distinctly different until convergence at metamorphic climax.

musculature, but not for masticatory musculature[38,39]. This study provides preliminary evidence for the existence of trait modules underlying carnivore development, but further investigations are necessary to substantiate trait modularity.

The developmental patterns suggest that the two phenotypes make different allocation decisions during the growth process, which inevitably results in developmental trade-offs. Carnivores shift growth from intestines to trophic structure, relative to omnivores, from the earliest stages of development. The intestine-length effect is striking because of its association with diet[40]. Long intestines are necessary to acquire resources from plant material and detritus (the typical tadpole diet), whereas shorter intestines are correlated with more carnivorous diets[22,40]. According to Pfennig[14], carnivores have shifted to a more carnivorous, proteinaceous diet in order to accelerate development, but although a higher-protein diet may speed development, increasing the chances of completing development in rapidly drying ephemeral ponds, the acceleration comes at the cost of reduced fat storage and smaller size at metamorphosis[14,29]. Metamorphosis is energetically taxing and Pfennig[14] has shown that omnivores are more likely than carnivores to survive through metamorphosis, presumably because of their greater fat stores. Small size at metamorphosis is correlated with many factors that may decrease fitness in postmetamorphic juvenile anurans, including increased risks of predation and desiccation (see discussion in [41]) and reduced oxygen consumption, endurance, hematocrit level, and heart size[42]. These factors may also be part of the cost of becoming a carnivore in spadefoot toads and should be addressed in future studies.

## ACKNOWLEDGMENTS

Many thanks are due to A.B. Thistle, D. Houle, A.A. Winn, T.F. Hansen, S.R. Storz, and three anonymous reviewers for comments and constructive criticism of the manuscript. This research was supported by the Theodore Roosevelt Memorial Fund (AMNH), Sigma Xi, and the Robert B. Short Fellowship (FSU). Collecting permit: Arizona SP710791.
